# Tuberculin Treatment in Sheffield

**Published:** 1913-08-16

**Authors:** J. Rennie

**Affiliations:** Medical Superintendent of the Sheffield Tuberculosis Dispensary.


### TUBERCULIN TREATMENT IN SHEFFIELD.

By Dr. J. RENNIE, Medical Superintendent of the Sheffield Tuberculosis Dispensary.

It is obvious that the economic importance of the tuberculin treatment of pulmonary tuberculosis is enormous if dispensary treatment with it is successful. If for four, six, or eight months' sanatorium treatment we can substitute a preliminary course of from six to eight weeks' life at a sanatorium, principally for its hygienic educational value as well as to increase the potential responsive power of the individual patient, then, if this can be followed by a period of eight to twelve months' tuberculin treatment as an out-patient at the tuberculosis dispensary, undergone while the greater number of these patients are following their employment and playing their part as useful citizens, the gain to the community will be enormous, not only from the financial standpoint but also from the point of view of that *bête noire* of sanatorium success—the after-care problem.

Such a scheme has been in vogue in Sheffield for about two years, where it was begun under Dr. John E. Chapman, now of the Local Government Board. Altogether, 353 patients have been under tuberculin treatment, and the records have been extremely encouraging as regards the immediate results, of which alone we can, of course, speak at present.

As there may always be raised the criticism of diagnostic error, or at least diverse opinion as to what signs and symptoms are sufficient to warrant the diagnosis of early phthisis, we have made an analysis of the records of fifty-two patients whose sputum at the time of commencement of treatment was found to contain tubercle bacilli, and who have finished a complete course of tuberculin treatment. The tuberculin administration has been in no sense haphazard. The tuberculins used, with the minimal and maximal doses, are noted in the table recording the results, and increase in dosage is usually in geometrical progression. There has been a continuity of treatment all through, and this definite system of dosage has been adhered to in so far as has been compatible with the individual treatment of the patient.

As there is still such a divergence of opinion as to what criteria should be accepted in order to gauge the success of any mode of treatment, we have preferred to submit a detailed account of the complete clinical records. Of the fifty-two patients under consideration thirty-five are males and seventeen females. Using Turban's classification, fourteen belong to Stage I., twenty-one to Stage II., and seventeen to Stage III. The average period of sanatorium residence was 8.2 weeks, while the average length of the tuberculin course extended over 12.2 months. We are safe in saying that all the cases except two showed considerable and often surprising improvement, both as regards general condition and physical signs.

Naturally, all the patients had gained considerable weight during their short sanatorium residence, but we find, on comparing their weights at the end of tuberculin treatment with those when they first came under observation, that there was then an average increase of 3.9 kilos.

As regards those showing an improved lung condition we have distinguished two classes. In those considered "Healed lesion" no adventitious sounds were to be detected, while we have classed as "Arrested disease" those chests which showed very considerable retrogressive changes with fibrosis. In corroboration of this we may say that forty-one of the patients after treatment had no sputum at all, or no tubercle bacilli could be found. Of the eleven still found with tubercle bacilli in their sputum, eight belonged to Stage III.

### Working Capacity.

All the cases belonging to Stages I. and II. were able to follow their full employment during the whole course of dispensary treatment, and, from recent inquiries, we find that this full capacity has been maintained. As regards cases belonging to Stage III., we looked on their treatment more in the nature of an experiment, and feel quite satisfied with the results. Eight of those seventeen patients before treatment were totally incapacitated. After the full course fourteen were then working, and the capacity of only one was totally impaired. We may say that we are still keeping these patients under supervision by their paying periodical visits to the dispensary.

We have also the records of other thirty-three cases who have completed treatment, but who, at the commencement, had either no sputum, or no tubercle bacilli could be found; nineteen, however, showed unequivocal signs of definite pulmonary lesion, while the remaining fourteen gave a positive focal reaction to a subcutaneous dose of not more than .01 of old tuberculin. Of these, only nineteen had educational sanatorium treatment, averaging nine weeks in duration, while the others were submitted solely to tuberculin treatment extending over a period averaging ten months. All except one showed at the end of treatment a complete absence of physical signs, while their general condition was excellent, and all are working. The average gain in weight of the adult patients in this class was 1.8 kilos. This smaller increase as compared with the gain in weight of those patients who had tubercle bacilli in their sputum is, no doubt, to be ascribed to their coming under treatment at an earlier stage, and consequently to their less impaired condition.

Of the others who were given a trial with tuberculin, thirteen showed no improvement, and therefore were considered unsuitable for further treatment. Five deaths have occurred, four as the result of an extension of the disease, although none had shown marked reactions, and one died suddenly four months after treatment—cause unknown. Twenty-three are "in suspense," some owing to want of interest or belief in the treatment, and others unable to attend at present owing to various temporary disabilities, work, etc. Eight have left the city.

The results have been so far encouraging that we have felt justified in giving the treatment a greatly extended trial. At present there are 199 cases under tuberculin at the dispensary, and in addition twenty definite tuberculous children at the Open Air Recovery School. Of the dispensary patients, 156 are full-wage earners, while the capacity of the remaining forty-three is more or less impaired. It is interesting to note that of 275 insured persons who have been notified as cases of pulmonary tuberculosis during the year ending July 13 last and who have applied for sanatorium benefit, no less than 115, or 42 per cent., have been considered suitable for treatment by the tuberculin method as described above. We are sure that it is only by similar extensive trials with carefully recorded results, conducted by different observers, that the intensive method of tuberculin treatment can be assessed at its proper value and be relegated to its proper place in therapeutic medicine.

